# Nursing Students' Perspectives Toward Providing Oral Health Care for Older People

**DOI:** 10.1155/ijod/5545284

**Published:** 2025-02-06

**Authors:** Arthi Veerasamy, Karl Lyons, Ian Crabtree, Jithendra Ratnayake, Paul Brunton

**Affiliations:** ^1^Faculty of Dentistry, University of Otago, Dunedin, New Zealand; ^2^School of Nursing, Otago Polytechnic, Dunedin, New Zealand; ^3^Curtin University, Bentley 6102, Western Australia, Australia

**Keywords:** geriatric oral health, oral health care in nursing, oral health education, oral health in long-term care, oral-systemic connection

## Abstract

**Introduction:** Oral care for older adults in residential homes and long-term hospital care has been largely ignored by health care professionals. The purpose of this study was to understand the perspectives of nursing students' on incorporating oral health care topics in the nursing curricula.

**Methods:** As a part of a broader cross-sectional quantitative study, we asked nursing students their opinions on incorporating an oral health care curriculum in their nursing programme. This resulted in a total of 148 responses, representing ~15% of New Zealand's nursing graduates and exceeding the anticipated survey response rate of 10%. The respondents were from 61% of nursing schools across the country. Thematic coding was used to analyze and report on the participants' responses.

**Results:** The nursing students thought oral health care education is overlooked in the nursing curriculum. Nursing students believed health professionals should be accountable for their patients' oral health.

**Conclusion:** Nursing students do not feel confident providing oral care for their patients. However, they recognize their responsibility and understand the need to provide oral health care as part of long-term care for older adults.

## 1. Introduction

Studies have shown an oral-systemic connection, and the impact of oral health is associated with dementia, cardiovascular conditions, diabetes, reduced life expectancy, and mortality. Indeed, a recent Cochrane database review by Hua et al. [1] suggested that proper oral hygiene care effectively prevents ventilated pneumonia, further strengthening the association between oral and general health.

In New Zealand, the percentage of people aged 65 and over is expected to increase up to 26% in 2043, up from 15% in 2016 [2, 3]. The prevalence of long-term conditions, multimorbidity, and polypharmacy increases with increasing age [4, 5], and nurses are expected to know the effect of drugs on health, including oral health and the association between oral and general health in addition to oral health care knowledge. Hence, caring for older people demands sophisticated oral health knowledge for health professionals, especially nurses.

Astvaldsdottir et al. [6] conducted a meta-analysis of systematic reviews and suggested that oral health care training for nursing staff could improve the overall care cost for older people. A study by Kelsen, Thomson, and Love [7] in New Zealand among nurses and care facilities managers indicated that more than half of residents needed assistance to perform their regular oral hygiene care, and dementia patients required further help. The participants identified that more training is needed for the staff, but they favored initiatives that did not require the facility to pay to train the staff rather than training the care facility staff and nurses working in long-term care facilities in hospitals, it would be more economical to prepare nursing students to care for the oral health of their future patients [7]. A study by Haresaku et al. [8] identified that improving oral health care knowledge in nursing undergraduate students has improved their oral health care practice, and also 95% of participants perceived that oral health care should be provided to older adults who are hospitalized and in residential home care. The role of nurses in geriatric patients' oral health has been well-documented in the literature [9–11].

The barriers to incorporating oral health care education in the nursing curriculum that have been identified in the literature are an already congested nursing curriculum, lack of experts available to contribute to the curriculum, and lack of awareness among policymakers such as accreditation bodies [12, 13].

Globally, integrating oral health into nursing education faces significant barriers that impede its adoption as a core component of curricula. A primary challenge is the limited expertise of nursing educators in oral health topics, resulting in a lack of confidence and capability to teach this area effectively [14]. Competing curriculum priorities often push oral health to the periphery, as nursing programs are already densely packed with content focused on general and specialized health care competencies [15]. Financial constraints further exacerbate the issue, as funding is often unavailable for developing and implementing oral health modules or interprofessional training involving dental professionals [16]. Moreover, there is a pervasive perception among educators and policymakers that oral health falls outside the scope of nursing practice, leading to its exclusion from educational frameworks [14]. Systemic issues, such as a lack of interprofessional collaboration between dental and nursing educators, contribute to stopping oral health teaching [17]. Cultural factors, including a lack of emphasis on oral health in certain regions, further hinder its integration [18, 19]. Overcoming these barriers requires a concerted global effort, including policy advocacy, increased funding, faculty development, and greater recognition of oral health as an integral component of overall health and nursing practice.

To date, nursing students' attitudes toward learning and caring for oral health in the older population have not been investigated. This study aimed to investigate nursing students' attitudes toward performing oral health care and learning oral health education in their nursing programme.

## 2. Methods

**Figure 1:**
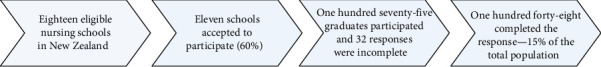
Recruitment process.

A qualitative approach was chosen to help understand nursing students' perspectives on oral health care for older adults in residential homes and hospital care. Data collection involved asking open questions as part of a broader study to identify final-year nursing students' views and thoughts on incorporating oral health care into the nursing curriculum and their experience learning about oral health in their nursing programme. To ensure that the study was valid, the criteria for credibility, transferability, dependability, and confirmability were followed [20]. Ethical approval for the study was obtained from the Human Ethics Committee, the University of Otago, and individual Institutional Review Boards of the Nursing Institutes in New Zealand (D19/264). Third-year nursing students were selected following a purposive sampling procedure because third-year students had experienced all 3 years of their nursing programme. At the time of the data collection, they had completed a clinical placement in both hospital care and residential care. It was anticipated that they would have more experience and knowledge regarding the topic than first- and second-year students. The population and sample size details are shown in Figure 1.

The survey package, which included a consent form, information sheet, and questionnaire on oral health care knowledge and participants' experience working with an older population, was sent to participants through an online platform (Moodle learning platform or course management system). Our original study questionnaire measured oral health knowledge, oral health care knowledge for older people, knowledge of the oral-systemic connections, and oral education practice characteristics in their institutes. This made the questionnaire too long for an online survey [21].

The research involved distributing a lengthy survey to nursing students, including two open-ended questions positioned at the end. Given the expected time and effort required to complete the survey thoroughly, it was essential to encourage participation and ensure a higher response rate. Additionally, the researchers recognized that ethical constraints in New Zealand nursing institutes limited direct contact with students. This necessitated reliance on the institute management or administration to distribute the survey links, potentially reducing engagement. The researchers introduced a reward system to address these challenges and motivate students to complete the entire survey. Two iPads were randomly awarded to participants who completed the survey, serving as an incentive to not only participate but also provide thoughtful and complete responses. This strategy was designed to enhance both the quantity and quality of the responses, ensuring valuable data for the study.

About 15% of 2020 students from 14 institutes participated in the survey. Out of 148 students, 90 students provided some opinion. The written comments as a part of the online survey were then analyzed. The authors in the current study had relevant research, educational, and teaching experience to contribute to the context of this study. The primary researcher Arthi Senthilkumar had experience in dental educational research, oral health literacy research, and qualitative research methodology. Author Karl Lyons had experience in gerodontolgy, dentistry, and prosthodontics and in addition, held a position as director of clinical education. Author Ian Crabtree held a position in the nursing institute as Head of School and had numerous years of teaching experience in nursing programmes and postgraduation qualification in nursing education. Author Paul Brunton held a position as a head of the Health Sciences Division which is New Zealand's leading provider of education and he contributed to the research base in primary oral health care, the biomedical sciences, and educational research.

**Figure 2:**
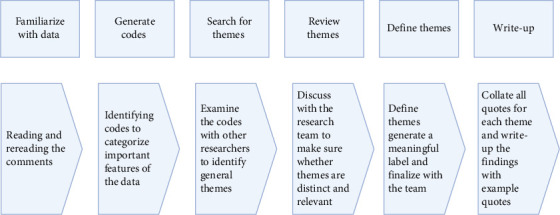
Process of thematic analysis.

A thematic analysis was undertaken utilizing the Braun and Clarke [22] analytical framework, as shown in Figure 2. A first analysis phase was conducted to understand the nursing students' opinions on incorporating oral health care knowledge education in the nursing curriculum. The raw quotes were organized and summarized into common themes, and data were interpreted under each theme and divided into subthemes or codes. The codes were generated during the analyses rather than being pregenerated. A second analysis phase was conducted to understand any new ideas not specific to the question asked, and codes were generated separately. Two new ideas identified while analyzing the data were students not being confident in performing oral care for older adults, and students thought health professionals should be accountable for oral health. Finally, five themes were identified by combining multiple codes from two phases of analysis.

### 2.1. Findings

When the comments were extracted from the survey, it was evident from the students' comments that there was a lack of oral health care education, and oral health care was widely ignored by health care professionals. Once the data saturation was confirmed, the decision to analyze and find the conclusion(s) was decided. The key themes that emerged were that “oral health education was very minimal,” “we regularly ignore oral health care of our patients,” “I do not feel confident in my oral health knowledge,” “health professionals are accountable for oral health,” and “oral health topic suggestions.”

### 2.2. Theme 1: Oral Health Education Is Very Minimal

The respondents signaled that there was minimal oral health education in their first year and later in their course oral health is largely ignored as nursing educators believe this is common sense.

“Extremely important but often is only touched on briefly. Not covered properly believing oral health care is common sense.”

“We were taught how to clean dentures and teeth but with no supporting knowledge.”

The majority of students indicated that there was extremely minimal discussion of oral health topics. However, the majority of students indicated that they had some oral health topic discussion in their first year. Participants consistently indicated that they needed more oral health education to care for older people.

“I can only remember one lecture given specifically about oral health in our first year.”

“We definitely need more than we have already.”

“It would be great to learn about it more to understand it properly and the importance of oral health.”

### 2.3. Theme 2: We Regularly Ignore Patient's Oral Health

The students strongly acknowledged that oral health has been consistently ignored in their nursing practice and education. They also signposted that the current nursing workforce does not consider oral health an important factor for patient care.

“Nurses in the workforce didn't seem to think that oral care was very important to worry about.”

The majority of participants indicated that they have experience working in residential homes and hospital care and oral health care is not in their care routine.

“I worked at residential home for a few years. We never ask in a hospital or care home if they need assistant with their oral care or remind them.”

“Oral health often gets missed in routine care and even doctors and specialists do not care or ask much about it.”

### 2.4. Theme 3: I Do Not Feel Confident in My Oral Health Knowledge

Nursing students suggested that they do not feel confident in oral health topics. Another important element that emerged was students indicated not feeling confident to care for their patients due to a lack of knowledge. They explained having a basic knowledge is not enough to care for their patient and comprehensive knowledge of oral health should be mandatory.

“It would be something good to touch on, so we have a basic knowledge around oral care in-depth.”

“It is not an area that I feel confident in my knowledge to care for my patients.”

### 2.5. Theme 4: Health Professionals Are Accountable for Oral Health

The majority of students indicated that nurses and health care professionals have a role in caring for patients' oral health, especially for frail and disabled older adults. They identified their role in caring for older adults' oral health and conceded that oral health care is a component of nursing care.

“Nursing requires taking care of patients' oral hygiene. It is an important part of nursing care.”

“Health professionals are accountable for the oral hygiene of those who are unable to do it themselves.”

### 2.6. Theme 5: Suggestion for Topic Areas

The students provided suggestions for oral health topic areas that could be incorporated into the nursing curriculum. There are various subthemes that emerged during analyses such as denture care, brushing techniques, and general health problems caused due to poor oral health, oral examination, and oral-systemic connection.

“How to perform oral exams”

“How to brush teeth properly, how to floss”

“Nursing students need to be trained more at noticing and interpreting oral discomfort and pain in non-verbal residents”

“A strong need for further education in all aspects of oral health as we do not have any.”

“Add impact of oral health on general health”

The majority of students suggested adding knowledge on denture care to nursing curriculum.

“How to clean dentures”

“Does a patient wearing denture need to clean their mouth?”

“Soaking dentures in water and sachet? How important it is to wash their teeth and dentures.”

## 3. Discussion

The study aimed to identify nursing students' opinions on incorporating oral health care education into the nursing curriculum. Findings suggested that oral health education is extremely minimal among nursing students. However, most students who responded to the study believed that oral health care education should be mandatory for nursing students. In addition, they signposted that oral health is an important component of nursing care, although it is widely ignored not only by nurses but also by other health care professionals.

The findings suggested that oral health topics are briefly covered in the first year of the nursing programme. The students thought it was not included in the curriculum as the educators believe oral health knowledge is common sense. The qualitative study conducted by Aljafari et al. [23] among parents of children undergoing tooth extraction under general anesthesia indicated that the parents knew oral hygiene and sugar limitation. However, further interviews suggested that they did not know the cariogenic effect of fruit juices. As indicated by the study participants in our study, an in-depth knowledge of oral health care is required to care for their patients.

A previous study has shown that the inability of nurses to perform an oral examination was a major barrier to providing dental care for their patients [24]. A recent study among nurses showed that nursing students' attitudes and confidence in performing an oral assessment significantly increased after providing a total of 2.5 h of oral health education in the first year and second year of their study [8]. In the current study, students indicated that having basic oral health knowledge was not enough, and they did not have confidence in performing oral health care for their patients. As shown in Haresaku et al.'s [8] study, increasing oral health care knowledge among nursing students does not need multiple hours of teaching, and appropriately designed content could be provided in minimal hours.

The findings of this study align with international literature, highlighting similar gaps in oral health education within nursing curricula. For instance, a study conducted in the United Kingdom reported that oral health is often perceived as a low-priority topic, with minimal inclusion in nursing programmes [25]. Similarly, research from the United States found that nursing students frequently graduate without adequate preparation to address oral health issues due to insufficient curriculum coverage [15]. While these challenges appear universal, context-specific barriers exist, such as limited access to oral health professionals in certain regions. New Zealand's situation reflects both global trends and unique local challenges, emphasizing the need for tailored strategies to improve oral health education within nursing programmes.

The role of health care professionals in oral health has been highly studied in the literature. The lack of training for physicians, nurses, and other health care workers is considered a barrier for them to providing oral health care and dental referrals for their patients [26], and oral health care education has historically been ignored in the medical curriculum [26]. In our study, students signaled the importance of a health care professional's role in providing oral health care for their patients, especially disabled patients.

Students suggested several oral health topics that should be included in the nursing curriculum, such as oral examinations, denture care, and the oral-systemic health connection. This recommendation is particularly timely given projections that edentulism in New Zealand will decrease fourfold between 2011 and 2031 [21]. As a result, the number of dentulous residents entering aged care facilities will rise, increasing the need for nurses to perform oral examinations on patients with fixed prostheses, such as crowns and implants. Nurses will also need to make appropriate and timely referrals to dental professionals to prevent and treat oral diseases.

Nursing curricula would benefit from integrating concise yet comprehensive oral health education modules to address these identified gaps. For example, 2–3 h of targeted instruction on oral examinations, denture care, and the oral-systemic health connection could significantly enhance students' knowledge and confidence. Practical training sessions, such as simulation exercises, would further facilitate skill development. Collaborating with oral health professionals could provide valuable interprofessional perspectives, enhancing the practical application of these topics. In line with this, the recent approval by the Dental Council of New Zealand to train oral health therapists and hygienists for adult care reinforces the importance of broadening oral health education in nursing. Policymakers might also consider appointing oral health professionals to work within residential care facilities, train, and support nurses in managing oral health care.

However, implementing these changes in the nursing curriculum will require careful planning, including leveraging available campus experts, resources, infrastructure, faculty appointments, administrative staff, and clinical facilities. Fortunately, the process of integrating oral health care into nursing curricula has already been successfully tested in several OECD countries, offering a roadmap for New Zealand to follow. For instance, two key programmes in the United States—the “Smile for Life” and “Train the Trainers” programmes under the Oral Health Nursing Education and Practice (OHNEP) initiative—serve as effective models for incorporating oral health education into nursing curricula [27]. Similarly, Japan's oral health education model, which focuses on concise, targeted training modules, has been effective in integrating oral health into broader health care education [28, 29]. These international examples provide valuable frameworks New Zealand can adapt to improve its nursing education programmes.

## 4. Conclusion

The findings of this study stressed the need for comprehensive oral health care education for nursing students. Nursing students do not feel confident about providing oral health care for their older patients. However, they believe that health care professionals should be accountable for caring for their older patients' oral health. New Zealand nursing educators could develop the oral health curriculum with the help of dental professionals to prevent and manage oral diseases in older adults in aged care facilities and long-term hospital care.

## Data Availability

The data underlying this article will be shared upon reasonable request from the corresponding author.
